# Effects of Notch signaling on the lineage commitment of human peripheral blood monocyte trilineage progenitor under inflammatory conditions

**DOI:** 10.1038/s41420-025-02807-z

**Published:** 2025-11-10

**Authors:** Sara Aničić, Maša Filipović, Ivo Krešić, Ozana Jakšić, Marta Radošević, Darja Flegar, Pavao Planinić, Marina Ikić Matijašević, Zrinka Jajić, Tomislav Kelava, Nataša Kovačić, Alan Šućur, Danka Grčević

**Affiliations:** 1 Laboratory for Molecular Immunology, Croatian Institute for Brain Research, University of Zagreb School of Medicine, Zagreb, Croatia; 2 Department of Physiology and Immunology, University of Zagreb School of Medicine, Zagreb, Croatia; 3 Department of Physiology, School of Medicine, University of Mostar, Mostar, Bosnia and Herzegovina; 4 Department of Clinical Immunology, Rheumatology, and Pulmonology, University Hospital “Sveti Duh”, Zagreb, Croatia; 5 Department of Rheumatology, Physical Medicine, and Rehabilitation, Clinical Hospital Center “Sestre Milosrdnice”, University of Zagreb School of Medicine, Zagreb, Croatia; 6 Department of Anatomy, University of Zagreb School of Medicine, Zagreb, Croatia

**Keywords:** Inflammation, Innate immunity, Osteoimmunology

## Abstract

Rheumatoid arthritis (RA) is a chronic autoimmune inflammatory disease, causing significant morbidity and disability. Inflammation-induced activation of myeloid cells is involved in disease pathogenesis and contributes to joint destruction. Due to the significant plasticity of myeloid lineage, peripheral blood (PBL) monocytes have the potential to differentiate into a variety of mature cells, including macrophages, osteoclasts, and dendritic cells (DCs), depending on the environmental cues and activated signaling pathways. Therefore, we aimed to determine how the Notch pathway affects differentiation of human PBL monocyte progenitor under inflammatory conditions. We first determined the frequency of monocyte subsets and identified a common trilineage monocyte progenitor (TMP), expressing the phenotype CD45^+^CD15^−^CD3^−^CD19^−^CD56^−^CD11b^+^CD14^+^, in the PBL of healthy controls and RA patients. To assess the effect of Notch-pathway activation on TMP differentiation, we then coated culture plates with the immobilized Notch ligands Jagged 1 (JAG1) and Delta-like ligand 1 (DLL1). Macrophages, osteoclasts, and DCs were differentiated from TMPs of control subjects by the appropriate cytokines (M-CSF, M-CSF/RANKL, or GM-CSF/IL4, respectively), whereas the addition of bacterial lipopolysaccharides mimicked an inflammatory environment. We observed that the TMP population is expanded in RA PBL and expresses Notch receptors, implicating its susceptibility to Notch regulation. Our results further suggest that in the context of inflammation, Notch signaling, especially via DLL1, polarizes TMP differentiation in favor of the pro-inflammatory and antigen-presenting capacity of DCs and macrophages, while suppressing phagocytosis and matrix degradation by macrophages and osteoclasts. Specifically, these Notch effects are seen as higher *IL1B* expression and enhanced T lymphocyte stimulation by DCs, higher HLA-DR expression and suppressed phagocytosis by macrophages, as well as lower *CTSK* expression and suppressed TRAP activity by osteoclasts. In conclusion, we demonstrated that the Notch-axis effectively regulates the commitment of common TMP into myeloid cell subtypes. Therefore, modulation of Notch signaling may be an important complementary approach to treating RA pathogenesis.

## Introduction

Myeloid lineage cells, such as monocytes, macrophages, dendritic cells (DCs), neutrophils, myeloid-derived suppressor cells, osteoclasts, or microglia, are important contributors to inflammatory and immune responses, but are also involved in processes of tissue homeostasis, cancer progression, bone resorption, self-tolerance, fibrogenesis, and repair [1–3]. Analogous to the well-described mouse tripotential progenitor, several studies agree on the existence of human bone marrow (BM) and peripheral blood (PBL) trilineage progenitor of osteoclasts, macrophages, and DCs [4–8]. Circulating common trilineage monocyte progenitors (TMPs) could arise from BM or be released from peripheral reservoirs, and thereafter be attracted to different tissues, exhibiting plasticity to differentiate into heterogeneous and functionally diverse populations of mature cells. Macrophages and DCs primarily serve in phagocytosis, antigen presentation, and cytokine secretion, whereas osteoclasts exclusively mediate bone resorption and contribute to bone remodeling [1, 9].

Monocytes can be aberrantly activated in different inflammatory conditions, including rheumatoid arthritis (RA), which is a chronic autoimmune disease affecting approximately 1% of the global population, characterized by systemic inflammation, joint infiltration by immune cells, and enhanced bone resorption [10–13]. Findings suggest that the percentage of circulating CD14^+^ monocytes is increased in RA, probably due to their greater recruitment and turnover in the inflamed joints [14, 15]. Once attracted to the target tissues, the TMP differentiation process is susceptible to environmental imprinting by surrounding cells, cytokines, and growth factors [16].

Given that all three cell types derived from TMP (osteoclasts, macrophages, and DCs) are responsive to the inflammatory environment in RA and significantly contribute to disease pathogenesis, targeting signals that modulate TMP commitment may be considered as a possible therapeutic approach. The Notch pathway is a ubiquitous juxtacrine form of intercellular communication that regulates cell self-renewal and differentiation of hematopoietic lineages. Notch-signalization participates in determining the cell fate during developmental processes and maintenance of tissue homeostasis in adult organisms [17]. In humans, canonical Notch-signaling occurs via the interaction of Notch ligands (Jagged (JAG) 1 and 2; Delta-like ligand (DLL) 1, 3, and 4) on the signal-sending cell, with the Notch receptors (Notch 1 to 4) on the adjacent signal-receiving cell, which results in the cleavage of the Notch intracellular domain (NICD) by the γ-secretase complex [18, 19]. Signaling continues by translocation of the NICD to the nucleus, displacement of transcriptional repressors, and association of the NICD with other transcriptional factors, resulting in the transcription of Notch canonical target genes, such as the HES and HEY families, as well as several lineage-specific genes involved in fate determination and function. Alternatively, NICD can remain in the cytoplasm to crosstalk with the components of other signaling pathways, such as Wnt, NF-κB, or Akt [9, 17].

The result of signaling through Notch is highly context-dependent, including effects of timing, location, and specific ligand/receptor interaction. The role of the Notch pathway is still inconclusive in myeloid lineage, especially in the context of human inflammatory diseases [20]. Therefore, we aimed to isolate PBL TMPs, expose them to Notch ligands in vitro, and assess their trilineage differentiation potential at the level of gene expression, phenotype profile, and functional properties. Our results suggest that under an inflammatory environment, Notch signaling, especially via DLL1, skews TMP differentiation toward DCs with the pro-inflammatory and immunostimulatory capacity, macrophages with suppressed phagocytosis but enhanced antigen-presenting potential, and osteoclasts with decreased activity and matrix-degrading ability.

## Results

### The frequency of TMPs within PBMCs was higher in RA compared to control subjects

**Fig. 1:**
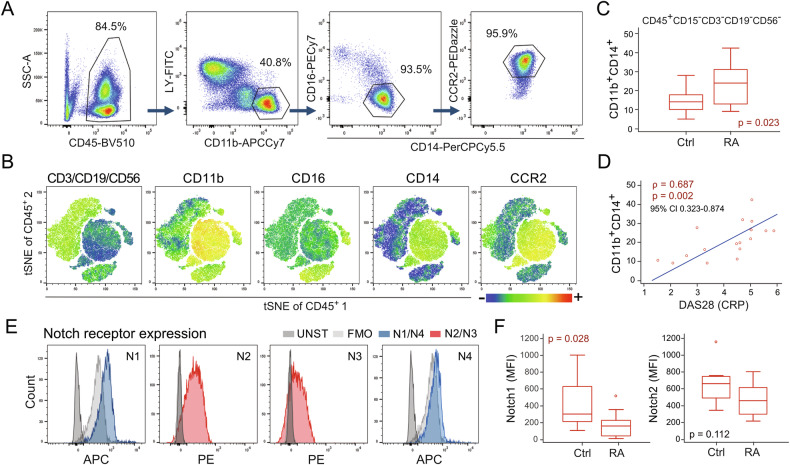
Flow-cytometric dissection of the common trilineage monocyte progenitor (TMP) in peripheral blood (PBL) samples of healthy controls (Ctrl) and patients with rheumatoid arthritis (RA). **A** Gating strategy for identification of the classical monocyte population in PBL. After delineation of live hematopoietic (CD45^+^) PBL cells not expressing granulocyte (CD15^−^) and lymphoid (LY; CD3^−^CD19^−^CD56^−^) markers, monocytes were identified by the expression of myeloid markers specific for classical inflammatory monocytes (CD11b^+^CD16^−^CD14^+^CCR2^+^) **B** Visual presentations of cell clusters performed by a T-distributed stochastic neighbor embedding (tSNE) algorithm using compensated fluorescence parameters for each marker (heatmap view of fluorescence intensity). **C** Frequencies of TMPs (CD15^−^CD45^+^CD3^−^CD19^−^CD56^−^CD11b^+^CD14^+^) in PBL samples of Ctrl subjects (*n* = 14) and RA patients (*n* = 26). **D** Association of the TMP frequency with RA disease activity score in 28 joints (DAS28) (*n* = 17). Individual values and trend lines are presented with Spearman’s rank correlation coefficient (ρ). Statistically significant difference was determined at *p* <0.05. **E** The expression of Notch receptors (N1 to 4) was evaluated in a defined TMP population. Representative histograms for Notch receptor expression are shown compared to unstained (UNST) and fluorescence-minus-one (FMO) controls. **F** Mean fluorescence intensity (MFI) of Notch1 and Notch2 expression on TMPs in Ctrl subjects (*n* = 10) and RA patients (*n* = 10). **C**, **F** Results are presented by box-and-whisker plot, horizontal lines represent the median, boxes represent the interquartile range (IQR), and whiskers represent 1.5 times the IQR. Effect size (*r*) was 0.30 to 0.49 (moderate effect). Statistically significant difference was determined at *p* <0.05; Mann–Whitney *U*-test.

The flow-cytometry analysis of monocyte subsets included delineation of live hematopoietic (CD45^+^) PBL cells not expressing granulocyte (CD15^−^) and lymphoid (CD3^−^CD19^−^CD56^−^) markers, followed by gating classical (CD11b^+^CD16^−^CD14^+^CCR2^+^), intermediate (CD11b^+^CD16^+^CD14^+^CCR2^lo^), and non-classical monocytes (CD11b^+^CD16^+^CD14^−^CCR2^−^) (Fig. 1A and Supplementary Fig. S1A). In line with our previous work [21, 22], we observed that a classical subset accounted for >90%, an intermediate subset for 3–4%, whereas a non-classical subset for only 1-2% of total CD11b^+^ monocytes (Fig. 1A and Supplementary Fig. S1A). The cell clusters presentation further confirmed simultaneous expression of CD11b, CD14, and CCR2 in approximately 30-40% of RA PBMCs (Fig. 1B). Based on phenotype profiling and subset abundancy, we further focused on CD15^−^CD45^+^CD3^−^CD19^−^CD56^−^CD11b^+^CD14^+^ cells as TMP population, which included classical and intermediate monocytes (expressing high and low levels of CCR2, respectively; Supplementary Fig. S1A). Compared to the controls, this population was significantly enlarged in RA and associated with the RA disease activity score DAS28 [23], calculated using C-reactive protein (CRP) (Fig. 1C, D). The same population expressed Notch receptors, showing the highest mean fluorescence intensity (MFI) of Notch2, moderate of Notch1, and, to a lesser extent, Notch3, and almost no signal of Notch4 (Fig. 1E). Interestingly, RA patients had a lower MFI of Notch1 expression compared to controls, indicating disease-related changes (Fig. 1F).

### TMP has trilineage potential to differentiate into macrophages, osteoclasts, and DCs

**Fig. 2:**
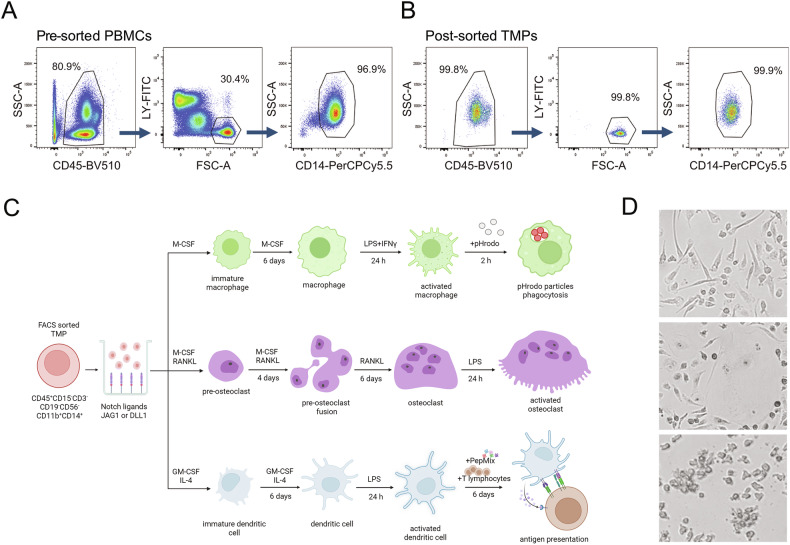
Differentiation potential of sorted trilineage monocyte progenitor (TMP) into macrophages, osteoclasts, and dendritic cells (DCs) under appropriate culture conditions. **A** The population of TMPs, defined as CD15^−^CD45^+^CD3^−^CD19^−^CD56^−^CD11b^+^CD14^+^ cells, was labeled and sorted from peripheral blood mononuclear cells (PBMCs) of control subjects. LY; CD3/CD19/CD56. **B** The re-analysis of sorted TMPs revealed sorting purity of target population above 99.5%. **C** Experimental design of trilineage differentiation. TMPs were seeded into plates with immobilized Notch ligands (JAG1 or DLL1). Cells were matured in the appropriate culture conditions, and LPS was added (last 24 h) to mimic inflammatory conditions. Functional maturation was assessed in a lineage-specific manner: macrophages were tested by the addition of bacterial particles to assess phagocytosis; osteoclasts were subjected to cytochemical staining for the activity of tartrate-resistant acid phosphatase; DCs were treated with peptide mix and co-cultured with autologous T lymphocytes to evaluate DC capacity to stimulate their proliferation. Created with BioRender.com **D** Representative brightfield images show typical morphology of differentiated macrophages, osteoclasts, and DCs derived from common TMP (top to bottom; magnification ×100).

Since the expression of Notch receptors seems to be altered in RA patients with established disease [20], we assumed that using RA cells could lead to incorrect interpretation of Notch stimulation and rather assessed trilineage differentiation in control subjects, with the addition of LPS to mimic the inflammatory environment. Highly efficient gating strategy and sorting protocol were applied to isolate CD15^−^CD45^+^CD3^−^CD19^−^CD56^−^CD11b^+^CD14^+^ cells, resulting in excellent population viability and purity (Fig. 2A, B and Supplementary Fig. S1B). Sorted cells, seeded into Notch ligand-coated wells under LPS stimulation (using wells coated with IgG anti-Fc and treated by LPS as controls), proved their TMP identity by efficiently differentiating into macrophages, osteoclasts, and DCs, acquiring the typical morphology of the respective lineage when cultured in the appropriate conditions (Fig. 2C). Mature macrophages were mainly elongated, firmly adherent mononuclear cells, whereas osteoclasts appeared as large, multinucleated cells with abundant cytoplasm. DCs became progressively less adherent, round cells with fine processes. For comparison, efficient trilineage differentiation was also shown for control differentiation (without LPS, IgG, or Notch ligands) and differentiation in wells coated with IgG anti-Fc (without LPS or Notch ligands) (Fig. 2D and Supplementary Fig. S2A). The effects of Notch-ligands were confirmed by increased expression of *HES1* (approximately 2–3 fold for JAG1 and 5–8 fold for DLL1; Supplementary Fig. S2B) and further assessed in TMP differentiation by lineage-specific genes, membrane marker profiling, and functional assays.

### Notch-signaling suppressed phagocytosis but stimulated antigen-presentation in macrophages

**Fig. 3:**
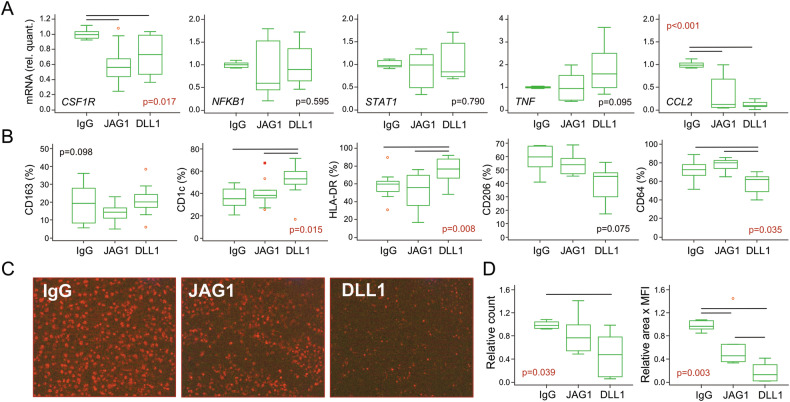
Characterization of macrophages differentiated from the trilineage monocyte progenitor (TMP) isolated from peripheral blood mononuclear cells (PBMCs) of control subjects. Using flow cytometry, TMPs were identified as CD15^−^CD45^+^CD3^−^CD19^−^CD56^−^CD11b^+^CD14^+^ cells, sorted, and differentiated into macrophages by adding M-CSF in wells coated with IgG anti-Fc only (IgG), or with additional treatment by Notch-ligand:Fc fusion proteins (JAG1 or DLL1). LPS and IFNγ were added 24 h before harvesting to induce the inflammatory response. **A** Gene expression in macrophages differentiated from TMPs was determined using TaqMan assays for macrophage-specific genes: *CSF1R*, *NFKB1*, *STAT1, TNF*, and *CCL2*, relative to the *HMBS* housekeeping gene and presented as normalized to the appropriate control wells (IgG anti-Fc without Notch-ligands) (*n* = 6 individual samples, 6 wells/sample). **B** The phenotype of macrophages differentiated from TMPs was determined using monoclonal antibodies specific for the macrophage lineage markers: CD163, CD1c, HLA-DR, CD206, and CD64 (*n* = 7 individual samples, 3 wells/sample). **C** Representative images and quantification of macrophage phagocytosis. Activation of rhodamine in acidic vesicles after phagocytosis was detected as pHrodo^+^ macrophages (magnification ×100). **D** Number and area × mean fluorescence intensity (MFI) of pHrodo^+^ macrophages were expressed per field of view and normalized to the appropriate control wells (IgG anti-Fc without Notch-ligands) (*n* = 4 individual samples, 2–3 wells/sample). Box-and-whisker plots: horizontal lines represent the median, boxes represent the interquartile range (IQR), and whiskers represent 1.5 times the IQR. Statistically significant difference was determined at *p* <0.05, Kruskal–Wallis with Conover post-hoc test.

Following TMP differentiation in M1-polarizing conditions [24] (M-CSF and LPS/IFNγ), macrophages fully matured into functional phagocytic cells. Their maturation and LPS/IFNγ-mediated polarization were associated with the increased expression of differentiation genes, such as *CSF1R*, *NFKB1*, and *STAT1*, compared to undifferentiated cells (day 0; Fig. 3A and Supplementary Fig. S3). However, we did not observe a significant Notch-impact on the expression of those genes, except for M-CSF receptor *CSF1R* (Fig. 3A). Addition of LPS and IFNγ downregulated *MERTK*, associated with M2-polarization [25], without changes by Notch ligands (Supplementary Figs. S3 and 4). In contrast, pro-inflammatory cytokine *TNF* was induced by LPS and IFNγ, with a trend of further increase by DLL1 (Fig. 3A and Supplementary Fig. S3). Finally, the chemokine *CCL2*, which can shape M1-macrophage polarization [26], was downregulated under Notch-stimulation (Fig. 3A). Flow-cytometric analysis did not show a Notch-induced difference in the expression of a scavenger receptor CD163 [27] (Fig. 3B) or lipid-presenting molecule CD1a [28] (Supplementary Fig. S4). However, expression of CD1c and HLA-DR, both involved in antigen presentation [29, 30], was increased under DLL1-stimulation (Fig. 3B). The same treatment decreased Fc-receptor CD64 and, to a lesser extent, mannose receptor CD206, associated with phagocytic activity [31]. Consistent with that, macrophage phagocytosis was significantly reduced under Notch-stimulation, as shown both by flow-cytometry (Supplementary Fig. S4B) and fluorescent cell imaging (Fig. 3C, D).

### Notch-signaling downregulated the expression of differentiation genes and TRAP-activity in osteoclasts

**Fig. 4:**
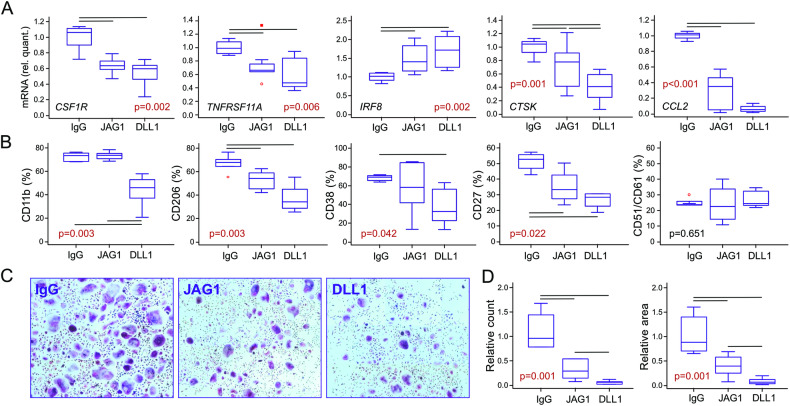
Characterization of osteoclasts differentiated from the trilineage monocyte progenitor (TMP) isolated from peripheral blood mononuclear cells (PBMCs) of control subjects. Using flow-cytometry, TMPs were identified as CD15^−^CD45^+^CD3^−^CD19^−^CD56^−^CD11b^+^CD14^+^ cells, sorted, and differentiated into osteoclasts by adding RANKL and M-CSF in wells coated with IgG anti-Fc only (IgG), or with additional treatment by Notch-ligand:Fc fusion proteins (JAG1 or DLL1). LPS was added 24 h before harvesting to induce the inflammatory response. **A** Gene expression in osteoclasts differentiated from TMPs was determined using TaqMan assays for osteoclast-specific genes: *CSF1R*, *TNFRSF11A*, *IRF8*, *CTSK*, and *CCL2*, relative to the *HMBS* housekeeping gene and presented as normalized to the appropriate control wells (IgG anti-Fc without Notch-ligands) (*n* = 5 individual samples, 6 wells/sample). **B** The phenotype of preosteoclasts differentiated from TMPs was determined using monoclonal antibodies specific for the osteoclast lineage markers: CD11b, CD206, CD38, CD27, and CD51/CD61 (n = 6 individual samples, 3 wells/sample). **C** Representative images and quantification of tartrate-resistant acid phosphatase (TRAP)-activity in osteoclasts. Expression of TRAP in multinucleated cells was used as a functional assay. **D** TRAP^+^ multinucleated cells with > 3 nuclei and a diameter ≥ 100 µm were counted as osteoclasts (magnification 100×). Number and area covered by large multinucleated TRAP^+^ osteoclasts were expressed per well and normalized to the appropriate control wells (IgG anti-Fc without Notch-ligands) (*n* = 4 individual samples, 3–4 wells/sample). Box-and-whisker plots: horizontal lines represent the median, boxes represent the interquartile range (IQR), and whiskers represent 1.5 times the IQR. Statistically significant difference was determined at *p* <0.05, Kruskal–Wallis with Conover post-hoc test.

Osteoclast maturation from TMPs, via M-CSF/RANKL and LPS, was confirmed by identifying large multinucleated cells with high TRAP-activity and induced differentiation genes *CSF1R*, *TNFRSF11A (RANK)*, *NFATC1*, and *CTSK* compared to undifferentiated cells (day 0; Fig. 4A and Supplementary Fig. S3). Both Notch ligands, JAG1 and DLL1, led to significant downregulation of chemokine *CCL2*, related to osteoclast activation [32], and several differentiation genes [33] (*CSF1R*, *TNFRSF11A, CTSK*), with no effect on *NFATC1* (Fig. 4A and Supplementary Fig. S5). In addition, the expression of *IRF8*, known to inhibit osteoclast formation [34], was enhanced by Notch-stimulation (Fig. 4A). Flow cytometry also showed several membrane markers to be downregulated by Notch-signaling, especially via DLL1 (Fig. 4B). This included adhesion molecule CD11b, mannose receptor CD206, adhesion/signal transduction molecule CD38, and TNF-family receptor CD27, all associated with enhanced osteoclast differentiation and activity [6, 35–37]. However, Notch stimulation did not significantly affect expression of integrin complex CD51/CD61, also known as vitronectin receptor [33]. Finally, evaluation of TRAP activity in mature osteoclasts further proved that Notch ligands significantly impaired their functional maturation (Fig. 4C, D).

### Notch-signaling enhanced pro-inflammatory properties and T lymphocyte stimulation by DCs

**Fig. 5:**
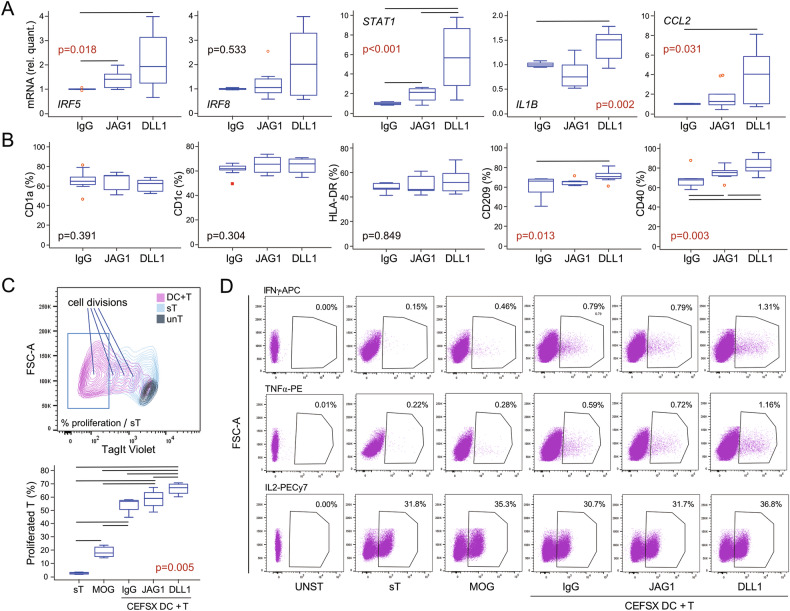
Characterization of dendritic cells (DCs) differentiated from the trilineage monocyte progenitor (TMP) isolated from peripheral blood mononuclear cells (PBMCs) of control subjects. Using flow-cytometry, TMPs were identified as CD15^−^CD45^+^CD3^−^CD19^−^CD56^−^CD11b^+^CD14^+^ cells, sorted, and differentiated into DCs by adding GM-CSF and IL4 in wells coated with IgG anti-Fc only (IgG) or with additional treatment with Notch-ligand:Fc fusion proteins (JAG1 or DLL1). LPS was added 24 h before harvesting to induce the inflammatory response. **A** Gene expression in DCs differentiated from TMPs was determined using TaqMan assays for DC-specific genes: *IRF5*, *IRF8*, *STAT1, IL1B*, and *CCL2*, relative to the *HMBS* housekeeping gene and presented as normalized to the appropriate control wells (IgG anti-Fc without Notch-ligands) (*n* = 5 individual samples, 6 wells/sample). **B** The phenotype of DCs differentiated from TMPs was determined using monoclonal antibodies specific for the DC lineage markers: CD1a, CD1c, HLA-DR, CD209, and CD40 (*n* = 6 individual samples, 3 wells/sample). **C** Stimulation of T lymphocyte proliferation was used as a DC functional assay. A representative plot shows fluorescence of proliferation dye TagIt Violet in unstimulated T lymphocytes (unT); T lymphocytes stimulated with anti-CD28/CD49d (sT); and T lymphocytes co-cultured with DCs (DC + T). Gates were set according to the proliferation rate in sT (upper panel). Quantification of proliferating T lymphocytes of sT or co-cultured DC + T: with negative control-peptides (MOG/DCs from IgG coated wells) or stimulating peptides (CEFSX/DCs from IgG or Notch-coated wells JAG1 or DLL1) (lower panel) (*n* = 4 individual samples, 3–4 wells/sample). Box-and-whisker plots: horizontal lines represent the median, boxes represent the interquartile range (IQR), and whiskers represent 1.5 times the IQR. Statistically significant difference was determined at *p* <0.05, Kruskal–Wallis with Conover post-hoc test. **D** Representative dot plots of T lymphocyte cytokine production: IFNγ, TNFα, and IL2 in sT and DC + T co-cultures (MOG/DCs from IgG coated wells; CEFSX/DCs from IgG, JAG1, or DLL1 coated wells), in comparison with unstained cells (UNST).

Under applied culture conditions (GM-CSF/IL4 and LPS), DCs were successfully differentiated from TMPs, showing morphological and functional properties of fully matured cells. Specifically, *IRF5* and *STAT6*, as DC-signature genes [38], were induced along with DC maturation (Fig. 5A and Supplementary Fig. S3). Moreover, Notch-signaling further increased the expression of *IRF5* (Fig. 5A). Expression of transcription factor *STAT1* and chemokine *CCL2*, regulating maturation of DCs [39–41], was also stimulated by Notch-signal, whereas induction of *IRF8* and *STAT6* did not reach a significant level (Fig. 5A and Supplementary Fig. S6). Finally, the gene expression of pro-inflammatory cytokine *IL1B* was significantly induced by DLL1. Flow-cytometric profiling revealed Notch-enhanced expression of costimulatory molecule CD40, which plays a crucial role in DC maturation, cytokine production, and T lymphocyte activation [42]. Several DC markers [38] (CD1a, CD1c, HLA-DR) were highly expressed on mature DCs, but Notch-stimulation did not further affect their expression level. In contrast, DLL1-signaling upregulated the expression of molecules involved in antigen processing, CD209 (DC-SIGN) and CD141 (Fig. 5B and Supplementary Fig. S6). DC functional assay was performed by adding a stimulating peptide mix (CEFSX) to the co-culture with autologous T lymphocytes, using T lymphocytes cultured without DCs (sT) or co-cultured with negative control-peptides (MOG) for comparison (Fig. 5C). In all these groups, addition of IL2/IL7/IL15 and co-stimulatory antibodies resulted in IL2 production by around 30% of proliferating T lymphocytes (Fig. 5D). In line with the expected frequency of specific T lymphocyte clones [43], around 1% of CEFSX-activated T lymphocytes secreted IFNγ and TNFα, compared to a lower percentage of cytokine-secreting T lymphocytes in control groups (sT and MOG) (Fig. 5D). However, only DCs matured under DLL1-stimulation showed enhanced ability to induce T lymphocyte proliferative response to CEFSX (Fig. 5C).

## Discussion

We aimed to reveal the Notch role in TMP commitment, differentiation, and function in the context of inflammation. In addition to regulating development, the Notch pathway is involved in the pathogenesis of different diseases, including some inflammatory states [9, 20, 44]. However, the cellular and molecular mechanisms by which Notch regulates innate immune cells are still unclear. In line with the studies by our group and others [14, 21, 22], we proved that a subset characterized by the CD45^+^CD15^−^CD3^−^CD19^−^CD56^−^CD11b^+^CD14^+^ phenotype (comprising classical and intermediate PBL monocytes) has trilineage differentiation potential, giving rise to functional macrophages, osteoclasts, and DCs. The population of TMPs is expanded in PBL of RA patients, showing a positive association with the disease activity score DAS28 and, at the same time, expresses Notch receptors, particularly Notch2 and, to a lesser extent, Notch1 and Notch3, making it responsive to Notch manipulation. A similar profile of Notch receptors was already observed on CD14^+^ monocytes, with the altered expression in the synovial tissue affected by arthritis [45–47]. We observed Notch1 downregulation on TMPs from RA patients, following the study showing reduced Notch1 and Notch3 expression in established compared to early RA [20]. Due to these RA-related changes in Notch receptor profile, we decided to study TMP differentiation under Notch stimulation in control (healthy) conditions, with the addition of LPS to mimic the inflammatory environment. We recognize that modulating Notch signaling in RA-derived cells from PBL and periarticular tissue would usefully complement our findings and plan to explore this subject in future research.

To assess TMP differentiation potential and functional properties, natural juxtacrine Notch signaling was modeled in vitro using immobilized Notch ligands under LPS treatment [48, 49]. Although Notch effects are highly context-dependent and difficult to model in vitro [50], we believe that by isolating human TMPs and exposing them to Notch ligands, we were able to reveal direct effects of Notch-pathway modulation without interference from other cells. In our experimental setting, Notch ligands significantly suppressed osteoclast TRAP activity and macrophage phagocytosis, while promoting the acquisition of the pro-inflammatory phenotype and antigen-presenting capacity in DCs and macrophages. This is particularly relevant for RA, given that all three lineages derived from TMP play a substantial role in RA pathophysiology. Circulating monocytes acquire an activated pro-inflammatory phenotype that contributes to inflammation and tissue destruction in RA [51]. In addition, inflammation enhances differentiation of osteoclasts, which mediate pathological bone destruction [20]. It has been shown in RA that Notch ligands JAG1 and JAG2, as well as DLL1 and DLL4, are upregulated on different cell types, including fibroblast-like synoviocytes, endothelial cells, and macrophages, which can shape the commitment of attracted monocytes through Notch receptors [52]. Moreover, Notch activation can induce these cells to secrete pro-inflammatory cytokines that drive RA pathology [51]. Notch target genes in primary human monocytes can be triggered by activation of Notch-signaling or through the TLR4 pathway, via NF-κB and MAPK, known to interact with Notch [9]. Conversely, it was reported that Notch-signal blockade via γ-secretase inhibitor DAPT interferes with LPS-mediated responses in monocyte lineage [53].

Our results showed that Notch ligands, especially DLL1, enhanced DC expression of *STAT1*, *IL1B*, CD209, and CD40 as well as DC ability to induce expansion of autologous T lymphocytes and their secretion of signature cytokines TNFα and IFNγ. These results align with a study showing that stimulation by JAG1 peptide promoted DC differentiation from adherent PBL cells, enhanced IL12 secretion, and induced allogeneic T lymphocyte proliferation, but reduced DC endocytic capacity [54]. Immobilized extracellular domain of DLL1 has been reported to promote monocyte maturation into DCs, while inhibiting their differentiation into macrophages [46]. Notch-associated transcriptional factor RBP-J (recombination signal binding protein for immunoglobulin κJ region) is involved in LPS responses in DCs, as its deletion in mice inhibits DC dendrite outgrowth and MHC-II expression [55]. A study by Liu et al. suggested that Notch ligands may differentially regulate terminal maturation of mouse DCs in vitro and during induced myelopoiesis in vivo, with DLL1 stimulating and JAG1 inhibiting this process through the opposite effect on Wnt signaling [56]. We proposed that DC stimulation by Notch ligands in our setting is probably achieved by enhanced expression of *IRF5* and, to a lesser extent, *IRF8*, in line with the study indicating that Notch regulates mouse BM DC generation by controlling IRF8 expression in an RBP-J-dependent manner [57]. CCR2/CCL2 axis may also contribute to the enhanced DC function [40], since DLL1 induced *CCL2* in DCs, in contrast to the opposite effects in macrophages and osteoclasts, showing lineage-specific regulation.

Notch activation suppressed macrophage CD64 expression and phagocytosis, while enhancing HLA-DR expression. In addition, CD1c, usually considered a DC marker, was also induced, in line with the study reporting that CD1c is expressed on a fraction of human PBL monocytes and mucosal macrophages under inflammatory conditions [29, 58]. Several other studies reported that Notch activation contributed to human macrophage polarization, in favor of M1- versus M2-type [59–62]. Specifically, DLL4 activated MAPK, NF-κB, and Akt pathways and transcription of pro-inflammatory genes [59]. On the contrary, Notch inhibition supported M2-polarization, suppressed pro-inflammatory cytokines (TNFα, IL6), and increased M2-markers CD206 and ARG1 [63, 64]. Although we applied M1-polarizing conditions (using LPS and IFNγ), resulting atypical phenotype (induced DC markers CD1c and HLA-DR, inconclusive changes in M1- (*CCL2* and *TNF*) and M2-markers (CD163 and CD206)) could be ascribed to the additional effect of Notch signaling. These findings are in line with the recent suggestion that plasticity of differentiating macrophages is far more complex, depending on the net effect of activated signaling pathways, giving rise to a variety of subtypes with specific phenotype and function, including M4-polarized macrophages with the pro-inflammatory properties and suppressed phagocytosis or DC-like subtype with enhanced expression of co-stimulatory molecules and pronounced antigen-presenting function [65].

Finally, Notch had significant suppressive effects on osteoclast differentiation, as we showed in the mouse model [49] and was observed in other studies [45, 66]. Notch ligands transcriptionally downregulated *CSF1R* and *TNFRSF11A*, receptors for the essential osteoclast-differentiation factors, as well as membrane markers (CD11b, CD206, CD38, CD27) associated with enhanced osteoclast activity [35–37]. In contrast to significant inhibition by DLL1 in our study, Sekine et al observed that JAG1/Notch1 inhibited but DLL1/Notch2 promoted osteoclastogenesis from mouse BM cells or human PBMCs [47]. Moreover, DLL1 blockade with neutralizing antibodies inhibited and, conversely, immobilized DLL1:Fc fusion protein enhanced osteoclastogenesis in vitro. Canalis et al further showed that the osteoclastogenic effect of Notch2 is mediated by HES1, through enhanced mitochondrial activity and metabolic processes [67, 68]. In contrast, constitutive Notch1 activation blocked osteoclast differentiation from human monocytes in vitro [69]. RBP-J (involved in Notch- and NF-κB-pathway) can suppress TNF-induced inflammatory osteoclastogenesis, with a minimal effect in homeostasis, whereas myeloid-specific deletion of RBP-J had an opposite effect [70, 71]. Osteoclast inhibition is believed to occur through RBP-J-induced suppression of NFATc1, while sustaining IRF8, known to block osteoclast differentiation [34, 70]. In our setting, we observed that DLL1 induced expression of both *HES1* and *IRF8*, with the final adverse effect on osteoclast activity, indicating that the regulation by Notch-pathway highly depends on context (homeostatic vs inflammatory), environment (autonomous vs indirect), stage (before vs after commitment) and, even, species (mouse vs human studies) used for experimental design [48, 49, 68, 72, 73].

In conclusion, our study suggests that the Notch pathway may act as a signaling switch for cell-autonomous modulation during differentiation from common TMP. This may substantially contribute to the pathogenesis of inflammatory diseases since these progenitors are increasingly released from BM and peripheral tissues, home to the affected sites, and acquire a differentiation fate depending on the environmental cues. In RA, abundance of DCs, macrophages, and osteoclasts in synovial tissue and subchondral bone correlates with disease severity, exacerbating joint inflammation and bone destruction [45, 66, 73]. With the limitation of being performed in vitro, our results support a potential role of the Notch pathway in myeloid plasticity during inflammation, which could be further explored for RA treatment strategies. Considering lineage-specific effects of Notch-signaling, delivery of Notch modulators using a nanoparticle system, engineered for targeted uptake by recognizing surface markers [74], or design of monoclonal antibodies to particular Notch ligand/receptor axis [44] may represent a viable approachs in chronic inflammatory diseases.

## Materials and methods

### Isolation of mononuclear cells from human blood

PBL samples (6–8 mL) were obtained by standard venipuncture from age-matched healthy adult donors and RA patients, recruited at the Clinical Hospital “Sveti Duh”, Clinical Hospital Center “Sestre Milosrdnice”, and University of Zagreb School of Medicine, following approval of the Ethics Committees and signing of the informed consent. Rheumatology specialists diagnosed RA patients (disease duration median 7 (interquartile range (IQR) 3–23) years; disease activity score in 28 joints (DAS28) median 4.6 (IQR 3.2–5.0)) according to the American College of Rheumatology criteria (ACR/EULAR 2010) [75, 76]. At the time of sampling, patients were undergoing treatment with conventional synthetic disease-modifying antirheumatic drugs (csDMARDs), with the exclusion of patients on biologic therapy. Control subjects were excluded if they had a history of immune, musculoskeletal, and connective tissue disorders, or active infectious diseases at the time of sampling.

PBL mononuclear cells (PBMCs) were isolated by density gradient using Histopaque (Sigma-Aldrich, St. Louis, USA), treated with Fc-block for 5 min at RT (BioLegend, San Diego, USA), and subsequently labeled for 30 min at 4 °C with monoclonal antibodies: panhematopoietic (CD45), granulocytic (CD15), lymphoid (CD3/CD19/CD56), and monocytic (CD14/CD16/CD11b/CCR2) (Supplementary Table S1). After exclusion of dead cells using DAPI (BioLegend), monocyte subsets (classical, intermediate, and non-classical) were compared between control (*n* = 14; 10 females, 4 males) and RA samples (*n* = 26; 20 females, 6 males) by flow-cytometry. In addition, the expression of Notch receptors was detected on a BD FACSAria IIu (BD Biosciences, Franklin Lakes, NJ, USA) and analyzed in FlowJo software (BD Biosciences), by setting gates according to unstained and fluorescence-minus-one (FMO) controls. A compensation matrix was established using single-stained cells for each fluorochrome.

### Activation of Notch-signaling using immobilized Notch ligands

Notch signaling was activated by coating culture plates with immobilized Notch ligands JAG1 and DLL1, using the modified protocol by Ashley et al and Filipović et al. [48, 49]. Briefly, 96-well plates were incubated overnight at 4 °C with rat-anti-human Fc IgG antibodies (BioLegend), at a concentration of 10 μg/mL in PBS, following by incubation with human fusion proteins JAG1:Fc or DLL1:Fc (R&D Systems, NE Minneapolis, MN, USA) at the concentrations of 10 μg/mL in PBS for 2 h at 37 °C, and subsequent washing with sterile PBS to remove unbound ligands. Notch activation was confirmed by the induced gene expression of *HES1* (Supplementary Table S2).

### Macrophage, osteoclast, and DC differentiation

PBMCs of healthy participants (*n* = 24) were treated by Fc-block (BioLegend), labeled with monoclonal antibodies (Supplementary Table S1), and subjected to fluorescence-activated cell sorting (FACS) for DAPI-negative TMP (CD15^−^CD45^+^CD3^−^CD19^−^CD56^−^CD11b^+^CD14^+^) isolation on a BD FACSAria IIu (BD Biosciences). Highly purified (>99.5% for all experiments) and viable ( >95% for all experiments) TMPs were then seeded in coated 96-well plates at a density of 2 × 10^4^ cells/well at 5% CO_2_ and 37 °C. Macrophages were differentiated in RPMI/10% FBS by 30 ng/mL recombinant human (rh) macrophage colony-stimulating factor (M-CSF; R&D Systems), osteoclasts in α-MEM/10% FBS by 15 ng/mL rhM-CSF (day 0–5; R&D Systems) and 100 ng/mL receptor activator of nuclear factor κB ligand (day 3–10; rhRANKL; R&D Systems), and DCs in RPMI/10% FBS by 75 ng/mL granulocyte-macrophage colony-stimulating factor (rhGM-CSF; Peprotech, Cranbury, USA) and 50 ng/mL interleukin 4 (rhIL4; R&D Systems). Media was changed every 2–3 days up to 7–10 days, and lipopolysaccharides (LPS) from *Escherichia (E.) coli* O111:B4 (Sigma-Aldrich) were added for the last 24 h (10 ng/mL LPS and 10 ng/mL interferon γ (rhIFNγ; Peprotech) for macrophages; 50 ng/mL LPS for osteoclasts; 100 ng/mL LPS for DCs) to activate differentiated cells [49].

### Phenotype profiling by flow cytometry

Cultured cells were harvested by a non-enzymatic detachment protocol (HBSS, 1% BSA, 1 mM EDTA in PBS, 50 µg DNAse II, 25 mM HEPES) [77]. Multicolor panels were used to label different lineages (for 30 min at 4 °C after Fc-block): CD163, CD1c, HLA-DR, CD206, CD64, and CD1a for macrophages; CD11b, CD206, CD38, CD27, CD51/CD61, and RANK for osteoclasts; CD1a, CD1c, HLA-DR, CD209, CD40, and CD141 for DCs (Supplementary Table S1). Stained cells were acquired on a BD FACSAria IIu (BD Biosciences) and analyzed in FlowJo software (BD Biosciences), by setting compensation matrix and gates according to unstained, single-stained, and FMO controls.

### RNA isolation and quantitative PCR gene expression analysis

RNA was extracted from cultured cells using TRI Reagent (Sigma Aldrich), converted to cDNA using reverse transcriptase (Applied Biosystems, Foster City, CA, USA), and amplified by quantitative (q)PCR using TaqMan Gene Expression Assays (Applied Biosystems) for the following genes: *CSF1R* (colony-stimulating factor 1 receptor, CD115, or M-CSF receptor), *NFKB1* (nuclear factor κB subunit 1), *STAT1* (signal transducer and activator of transcription 1), *STAT6*, *MERTK* (MER proto-oncogene, tyrosine kinase), *CCL2* (C-C motif chemokine ligand 2), *TNFRSF11A* (receptor RANK), *NFATC1* (nuclear factor of activated T cells 1), *IRF5* (interferon regulatory factor 5), *IRF8*, *CTSK* (cathepsin K), *IL1B* (interleukin 1β), *TNF* (tumor necrosis factor α) (Supplementary Table S2). Gene expression was presented as RNA relative quantity after normalization to the expression level of a housekeeping gene *HMBS* (hydroxymethylbilane synthase), as described previously [49].

### Osteoclast TRAP-activity

At osteoclast culture day 9-10, cells were fixed with 4% paraformaldehyde and subsequently stained for tartrate-resistant acid phosphatase (TRAP)-activity according to the manufacturer’s instructions (Leukocyte acid phosphatase kit; Sigma-Aldrich). Osteoclasts were visualized with an Axiovert 200 light microscope (Carl Zeiss Microscopy, Jena, Germany), identified as TRAP-positive multinucleated cells (>3 nuclei, diameter ≥ 100 µm), and quantified using FIJI (ImageJ) image analysis software, as described previously [21, 49].

### Macrophage phagocytosis assay

At macrophage culture day 7, a phagocytosis assay was performed using pHrodo *E. coli* particles (Invitrogen, Thermo Fisher Scientific, Waltham, MA, USA), following the manufacturer’s instructions. Briefly, after incubation with *E. coli* particles at 37°C for 2 h, fluorescent imaging was conducted to capture red fluorescence specifically emitted by the acidified, phagocytosed particles (Axiovert 200, Carl Zeiss). Quantifying fluorescent signal intensity and area was performed using FIJI (ImageJ) image analysis software, as described previously [49].

### DC antigen presentation

At culture day 7, DCs were transferred to U-bottom 96-well plates, co-cultured for 6 days with autologous T lymphocytes (at a 1:4 ratio) labeled with TagIt Violet proliferation dye (1:1000 dilution; BioLegend) in RPMI/10% FBS, supplemented with 20 IU/mL IL2, 20 ng/mL IL7, and 20 ng/mL IL15 (PeproTech), and stimulated with 5 µg/mL CEFSX Ultra SuperStim Pool (stimulating peptide pool; JPT Peptide Technologies, Berlin, Germany). For the final 18 h, cells were re-stimulated with the CEFSX peptide pool in the presence of co-stimulatory antibodies against CD28/CD49d (2 µg/mL each; BioLegend), according to the modified protocol by Cimen Bozkus et al. [78]. Wells containing only unstimulated T lymphocytes (unT); only T lymphocytes cultured with IL2/IL7/IL15 and co-stimulated with anti-CD28/CD49d (sT); or co-cultured cells (DCs and autologous T lymphocytes) treated in the same way as described above, except using negative control-peptides derived from a myelin oligodendrocyte glycoprotein (MOG; JPT Peptide Technologies) instead of CEFSX, were used for comparison. To detect cytokine production, cells were concurrently treated (final 16 h) with protein transport inhibitors, brefeldin A/monensin (5 µg/mL and 2 µM, respectively; BioLegend). Following re-stimulation, cells were treated with Fc-block (BioLegend), labeled with Zombie viability dye (1:500 dilution; BioLegend), fixed and permeabilized (Fixation Buffer, Intracellular Staining Permeabilization Wash Buffer; BioLegend), labeled using anti-IFNγ, anti-TNFα, and anti-IL2 antibodies (Supplementary Table S1), assessed by flow-cytometry on a BD FACSAria IIu (BD Biosciences), and analyzed using FlowJo software (BD Biosciences).

### Statistical analysis

The results were statistically analyzed and plotted using MedCalc (MedCalc Statistical Software version 13.1.2, Ostend, Belgium). Data distribution normality was assessed using the Kolmogorov–Smirnov test. Results are presented as medians with IQR and plotted as box-and-whisker diagrams. For some measurements (indicated in figure legends), values were normalized to the control group. Differences between groups were analyzed by the Mann-Whitney U-test or the Kruskal–Wallis test, followed by the Conover test for group-to-group comparisons. Effect size (r) for comparisons between controls and RA patients was calculated by dividing the z-statistic by the square root of the total sample size. Based on our previous studies [21, 49], the number of included subjects would allow the detection of a moderate effect size. Associations between variables were assessed by Spearman’s rank correlation. Statistical significance was set to α <0.05.

## Supplementary information

Suppl Fig S1

Suppl Fig S2

Suppl Fig S3

Suppl Fig S4

Suppl Fig S5

Suppl Fig S6

Suppl Table S1

Suppl Table S2

## Data Availability

The corresponding authors will provide the original data used to support the findings of this study upon reasonable request.
