# Supplementary figures and images for: Effects of Notch signaling on the lineage commitment of human peripheral blood monocyte trilineage progenitor under inflammatory conditions
Source: Cell Death Discov. 2025 Nov 10;11:519. doi: 10.1038/s41420-025-02807-z (PMC12602708; doi:10.1038/s41420-025-02807-z)

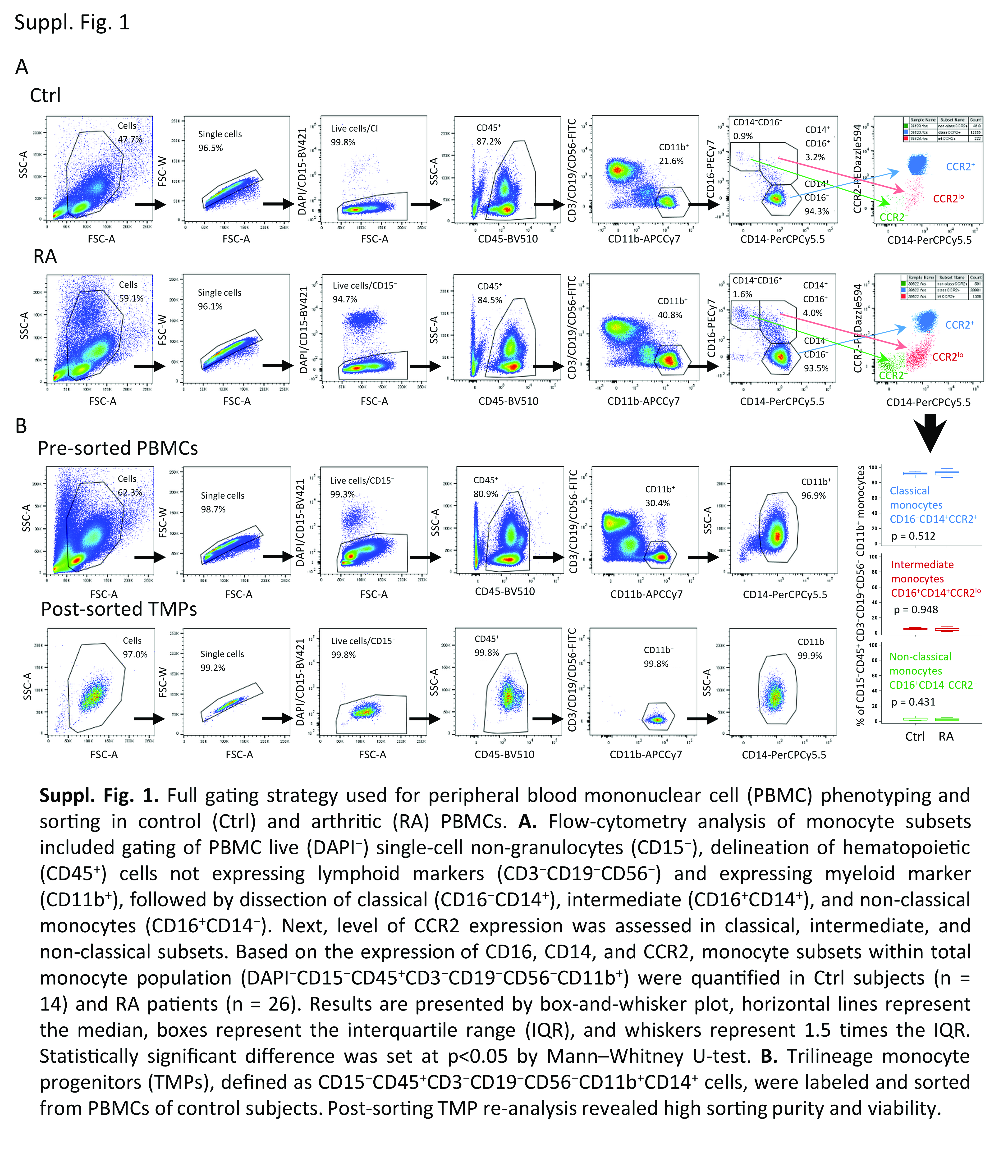

Supplement: Supplementary file 1 — Suppl Fig S1 [file 41420_2025_2807_MOESM1_ESM.tif]

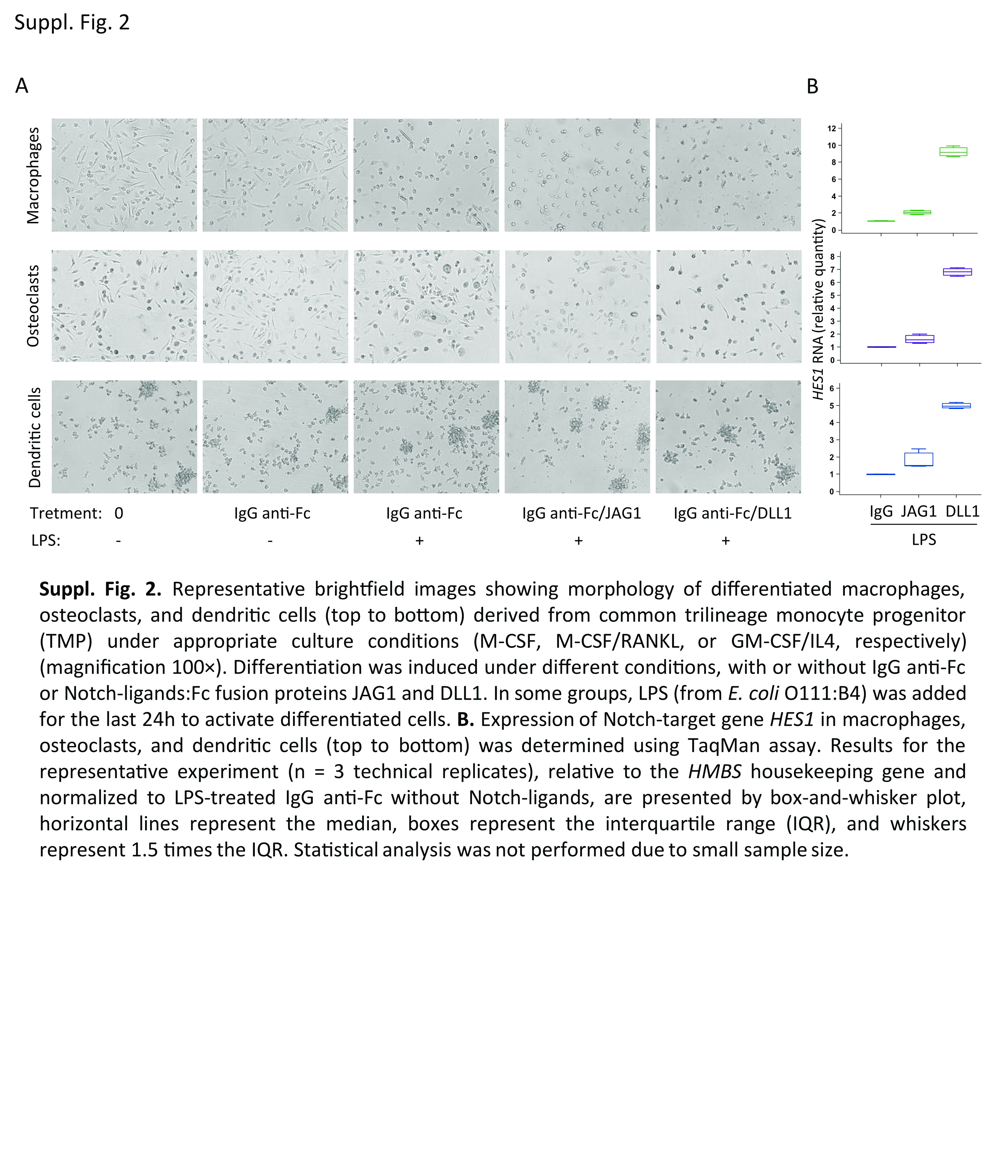

Supplement: Supplementary file 2 — Suppl Fig S2 [file 41420_2025_2807_MOESM2_ESM.tif]

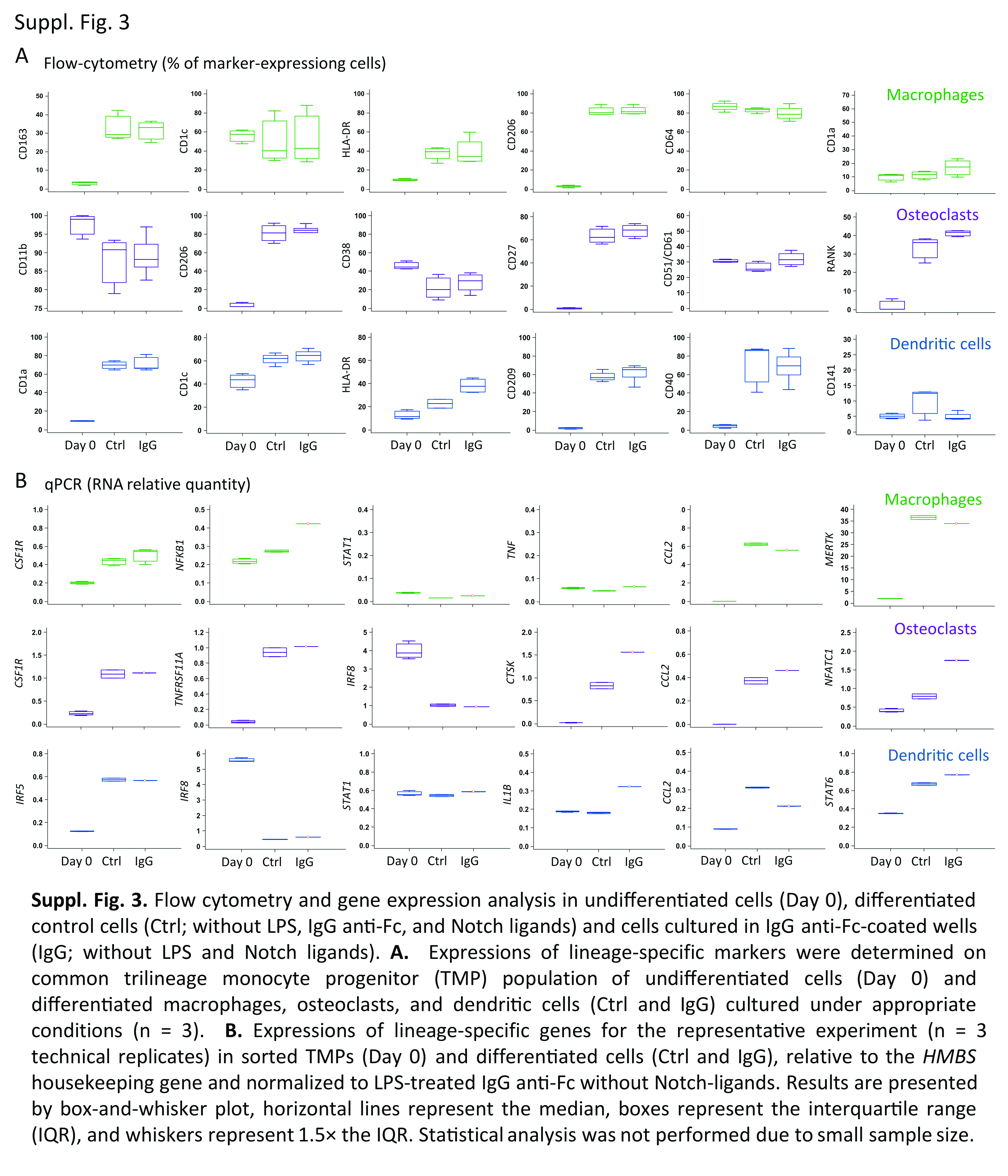

Supplement: Supplementary file 3 — Suppl Fig S3 [file 41420_2025_2807_MOESM3_ESM.tif]

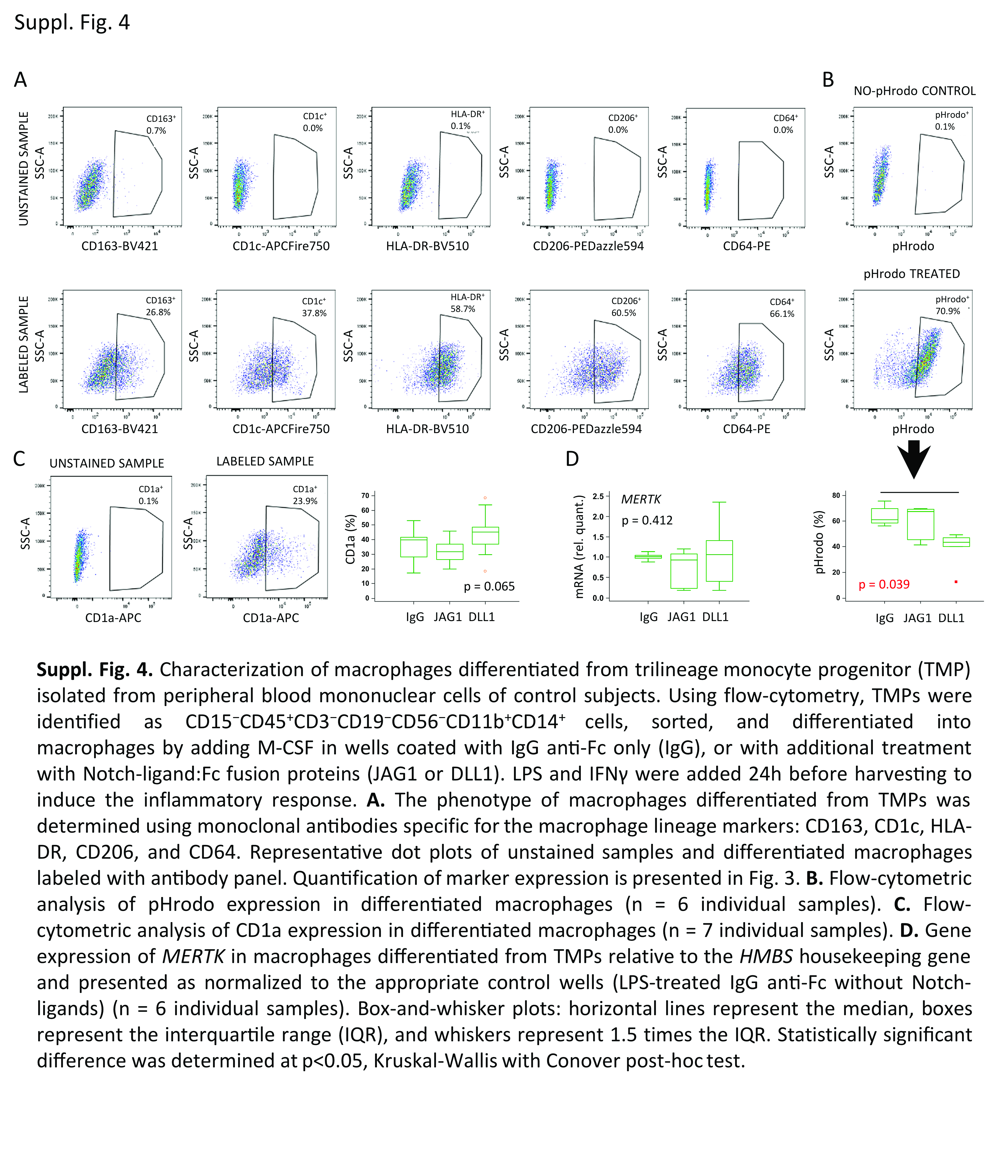

Supplement: Supplementary file 4 — Suppl Fig S4 [file 41420_2025_2807_MOESM4_ESM.tif]

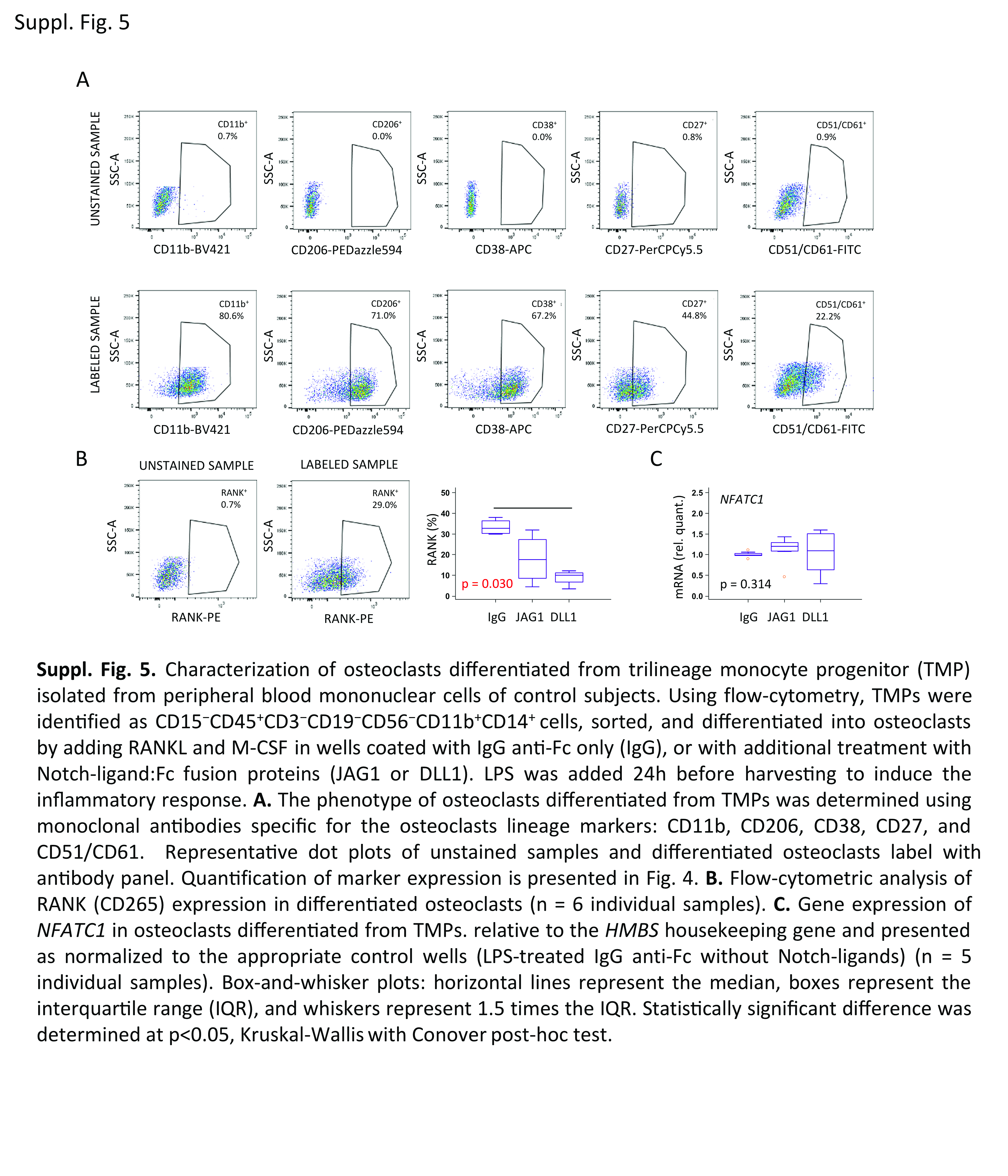

Supplement: Supplementary file 5 — Suppl Fig S5 [file 41420_2025_2807_MOESM5_ESM.tif]

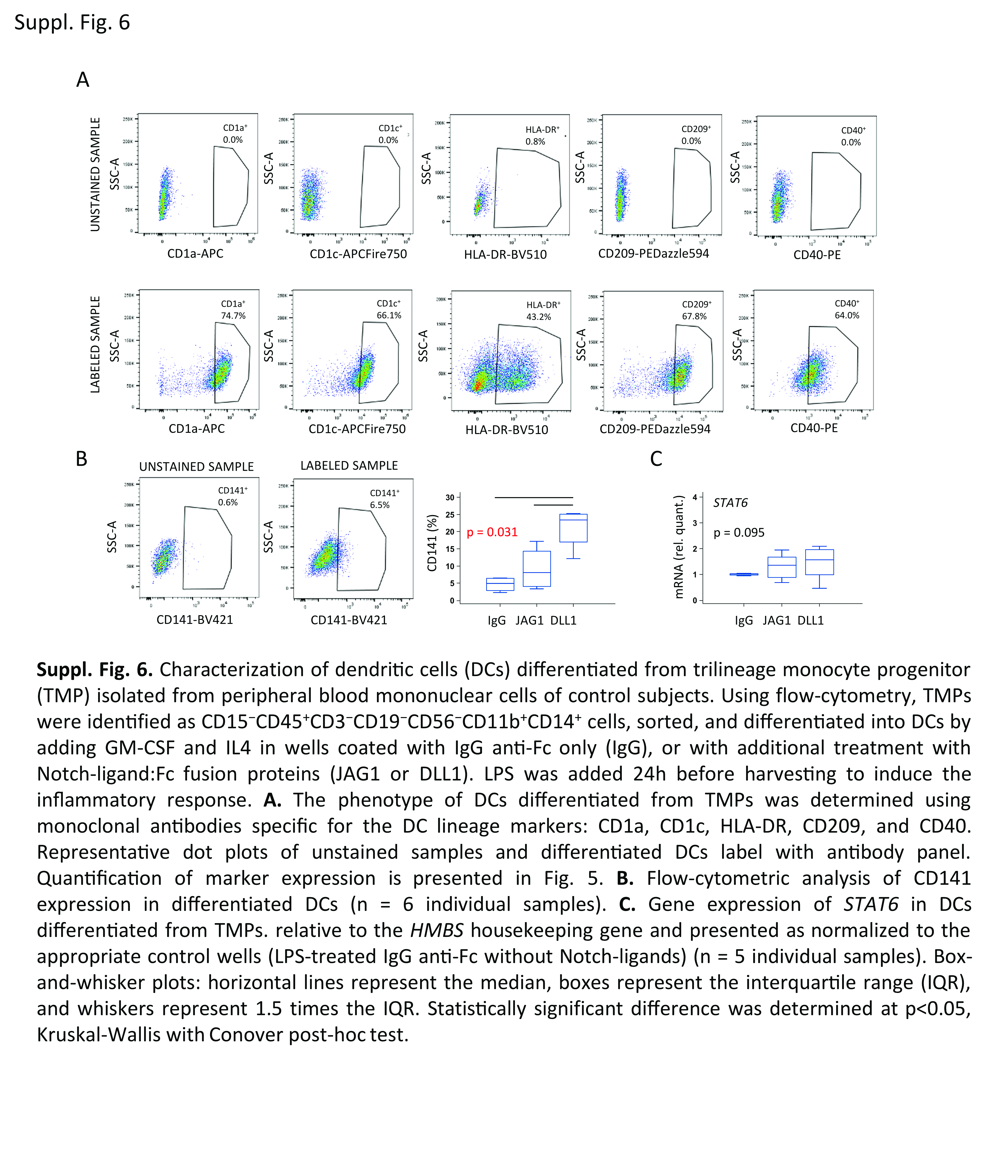

Supplement: Supplementary file 6 — Suppl Fig S6 [file 41420_2025_2807_MOESM6_ESM.tif]
