# Supplementary material for: Effects of Notch signaling on the lineage commitment of human peripheral blood monocyte trilineage progenitor under inflammatory conditions
Source: Cell Death Discov. 2025 Nov 10;11:519. doi: 10.1038/s41420-025-02807-z (PMC12602708; doi:10.1038/s41420-025-02807-z)
Supplement: Supplementary file 7 — Suppl Table S1 [file 41420_2025_2807_MOESM7_ESM.docx]

| **Marker** | **Clone** | **Conjugate** | **Manufacturer** |
| --- | --- | --- | --- |
| CD45 | HI30 | BV510 | BioLegend, San Diego, CA, USA |
| CD15 | W6D3 | BV421 | BioLegend, San Diego, CA, USA |
| CD3 | OKT3 | FITC | BioLegend, San Diego, CA, USA |
| CD19 | SJ25C1 | FITC | BioLegend, San Diego, CA, USA |
| CD56 | QA17A16 | FITC | BioLegend, San Diego, CA, USA |
| CD14 | HDC14 | PerCPCy5.5 | BioLegend, San Diego, CA, USA |
| CD14 | 63D3 | PECy7 | BioLegend, San Diego, CA, USA |
| CD16 | B73.1 | PECy7 | BioLegend, San Diego, CA, USA |
| CD11b | ICRF44 | APCCy7 | BioLegend, San Diego, CA, USA |
| CD11b | M1/70 | eFluor450; PECy7 | BioLegend, San Diego, CA, USA |
| CD1a | HI149 | APC | BioLegend, San Diego, CA, USA |
| CD1c | L161 | APCFire750 | BioLegend, San Diego, CA, USA |
| CD209 | 9E9A8 | PEDazzle594 | BioLegend, San Diego, CA, USA |
| HLA-DR | L234 | BV510 | BioLegend, San Diego, CA, USA |
| CD40 | 5C3 | PE | BioLegend, San Diego, CA, USA |
| CD163 | GHI/61 | BV421 | BioLegend, San Diego, CA, USA |
| RANK | 80704 | PE | R&D Systems, BioTechne, Abingdon, UK |
| CD51/CD61 | 23C6 | FITC | eBioscience, Thermo Fisher Sci., Waltham, MA, USA |
| CD206 | 15-2 | PEDazzle594 | BioLegend, San Diego, CA, USA |
| IFNγ | B27 | APC | BioLegend, San Diego, CA, USA |
| IL2 | MQ1-17H12 | PECy7 | BioLegend, San Diego, CA, USA |
| TNFα | Mab11 | PE | BioLegend, San Diego, CA, USA |
| CD141 | M80 | BV421 | BioLegend, San Diego, CA, USA |
| CD80 | 2D10 | BV510 | BioLegend, San Diego, CA, USA |
| CD64 | 10.1 | PE | BioLegend, San Diego, CA, USA |
| CD27 | M-T271 | PerCPCy5.5 | BioLegend, San Diego, CA, USA |
| Notch1 | REA849 | APC | Miltenyi Biotec, Bergisch Gladbach, Germany |
| Notch2 | REA1204 | PE | Miltenyi Biotec, Bergisch Gladbach, Germany |
| Notch3 | REA1219 | PE | Miltenyi Biotec, Bergisch Gladbach, Germany |
| Notch4 | MHN4-2 | APC | BioLegend, San Diego, CA, USA |
| CD28 | 28.2 | purified | BioLegend, San Diego, CA, USA |
| CD49d | 9F10 | purified | BioLegend, San Diego, CA, USA |

**Table S1. Monoclonal antibodies used for flow cytometry and *in vitro* treatment**
