# Supplementary material for: Effects of Notch signaling on the lineage commitment of human peripheral blood monocyte trilineage progenitor under inflammatory conditions
Source: Cell Death Discov. 2025 Nov 10;11:519. doi: 10.1038/s41420-025-02807-z (PMC12602708; doi:10.1038/s41420-025-02807-z)
Supplement: Supplementary file 8 — Suppl Table S2 [file 41420_2025_2807_MOESM8_ESM.docx]

**Table S2. TaqMan Gene Expression Assays used for quantitative PCR analysis**

| **Gene** | **Protein Coding** | **Assay ID** | **Manufacturer** |
| --- | --- | --- | --- |
| *CSF1R* | CD115, M-CSF receptor, cFms | Hs00911250_m1 | Applied Biosystems, Thermo Fisher Scientific, Waltham, MA, USA |
| *NFKB1* | nuclear factor κB subunit 1 | Hs00765730_m1 | Applied Biosystems, Thermo Fisher Scientific, Waltham, MA, USA |
| *STAT1* | signal transducer and activator of transcription 1 | Hs01013996_m1 | Applied Biosystems, Thermo Fisher Scientific, Waltham, MA, USA |
| *STAT6* | signal transducer and activator of transcription 6 | Hs00598625_m1 | Applied Biosystems, Thermo Fisher Scientific, Waltham, MA, USA |
| *MERTK* | MER proto-oncogene, tyrosine kinase | Hs01031979_m1 | Applied Biosystems, Thermo Fisher Scientific, Waltham, MA, USA |
| *CCL2* | C-C motif chemokine ligand 2 | Hs00234140_m1 | Applied Biosystems, Thermo Fisher Scientific, Waltham, MA, USA |
| *TNFRSF11A* | receptor activator of NF-κB (RANK) | Hs00187189_m1 | Applied Biosystems, Thermo Fisher Scientific, Waltham, MA, USA |
| *NFATC1* | nuclear factor of activated T cells 1 | Hs00542675_m1 | Applied Biosystems, Thermo Fisher Scientific, Waltham, MA, USA |
| *IRF5* | interferon regulatory factor 5 | Hs00158114_m1 | Applied Biosystems, Thermo Fisher Scientific, Waltham, MA, USA |
| *IRF8* | interferon regulatory factor 8 | Hs00175238_m1 | Applied Biosystems, Thermo Fisher Scientific, Waltham, MA, USA |
| *CTSK* | cathepsin K | Hs00166156_m1 | Applied Biosystems, Thermo Fisher Scientific, Waltham, MA, USA |
| *IL1B* | interleukin 1β | Hs01555410_m1 | Applied Biosystems, Thermo Fisher Scientific, Waltham, MA, USA |
| *TNF* | tumor necrosis factor α | Hs99999043_m1 | Applied Biosystems, Thermo Fisher Scientific, Waltham, MA, USA |
| *HES1* | hairy and enhancer of split-1, transcription factor | Hs00172878_m1 | Applied Biosystems, Thermo Fisher Scientific, Waltham, MA, USA |
| *HMBS* | hydroxymethylbilane synthase | Hs00609296_g1 | Applied Biosystems, Thermo Fisher Scientific, Waltham, MA, USA |
